# Mental Health Service Use among Older Adults at Risk of Suicide

**DOI:** 10.1093/geroni/igaf122.3053

**Published:** 2025-12-31

**Authors:** Afroze Shaikh

**Affiliations:** The University of Texas at Austin, Austin, Texas, United States

## Abstract

Older adults present with historically high rates of suicide (CDC, 2023), yet little research has examined the help-seeking behavior of older adults at elevated risk (Wang et al., 2023). Recent changes in Medicare policy also allow for licensed professional counselors (LPCs) to serve as Medicare providers, potentially increasing access to mental health care for older adults. This study, therefore, sought to examine predictors of proximal suicide risk factors (e.g., thwarted belongingness and perceived burdensomeness; Van Orden et al., 2012) among a national sample of older adults (n = 806) and factors that influence mental health service use among those at elevated risk of suicide using the Andersen Behavioral Model of Health Service Use (Andersen, 1995). Predictors of proximal suicide risk factors included difficulty accessing medical care and lower incomes. We found that 370 participants met clinical cutoff scores for elevated suicide risk (Mitchell et al., 2017) and that only 12.7% (n = 47) reported service use for mental health concerns within the past year, whereas only 2 (0.54%) reported service use for suicide concerns. Among the factors that influenced the need for services, greater levels of suicide behavior (OR = 1.191, p = .009), pain-related emotional burden (OR = 1.078, p = .004), and psychological distress (OR = 1.101, p = .011) increased the likelihood of mental health service use over the past year. Implications to promote belongingness and increase mental health service use among older adults will be discussed.

